# *LIN28B* Polymorphisms Confer a Higher Postoperative Recurrence Risk in Reproductive-Age Women with Endometrial Polyps

**DOI:** 10.1155/2022/4824357

**Published:** 2022-02-27

**Authors:** Mei-Yin Lu, Xiao-Hong Li, Jia-Li Niu, Bin Liu

**Affiliations:** ^1^Department of Biobank, Shenzhen Baoan Women's and Children's Hospital, Jinan University, Shenzhen, Guangdong, China; ^2^Department of Reproductive Health, Shenzhen Baoan Women's and Children's Hospital, Jinan University, Shenzhen, Guangdong, China

## Abstract

The RNA-binding protein LIN28B is an important factor for cell proliferation. Because *LIN28B* polymorphisms have been shown to be relative with the recurrence of some hyperplastic diseases, we hypothesized that genetic variants of *LIN28B* gene were associated with postoperative recurrence risk in reproductive-age women with endometrial polyps (EP). In a hospital-based cohort of 351 reproductive female patients underwent hysteroscopic polypectomies between May 2018 and Jan 2020, we genotyped two common polymorphisms in *LIN28B* gene (rs369065 C > T and rs314280 A > G) and analyzed their associations with the risk of postoperative recurrence in multiple Cox regression model. When followed up to Jun 2021, carries of rs369065 TT genotype had an increased risk of polyp recurrence (adjusting hazard ratio [HR] = 1.883, 95% confidence interval [CI] = 1.033 − 3.434) and had a shorter time to recurrence (median time 352 vs. 342 days, log-rank *P* < 0.01), compared to the CC/CT genotype. Further stratification analysis showed that the increased risk of rs369065 TT genotype was more evident in patients who were older than 33 years (adjusted HR = 2.597, 95%CI = 1.037 − 6.505), had a single polyp (adjusted HR = 2.545, 95%CI = 1.059 − 6.113), and had smaller polyps (<1.2 cm, adjusted HR = 2.708, 95%CI = 1.042 − 7.043). However, no significant association between rs314280 A > G polymorphism and the risk of polyp recurrence was found. Our study suggests that rs369065 TT genotype of *LIN28B* gene is associated with an increased postoperative recurrence risk in EP patients, especially in those with fewer and smaller polyps. These findings implicate a precise choice of clinical counseling and decision making. Larger studies in different ethnic populations are warranted.

## 1. Introduction

Endometrial polyp (EP) is a focal hyperplasia of endometrial basal layer, composing of endometrial glands, vessels, and nearby stroma [1]. It is a common gynecologic disease with higher prevalence in infertile women [2]. Hysteroscopic polypectomy is commonly used to treat EP in infertile patients, which can improve spontaneous conception as well as assisted reproduction [2–4]. However, the rate of polyp recurrence after polypectomy remains 2.5 ~ 44%, which hinders the improvement of fertility in these patients [2–5]. Therefore, postoperative recurrence of EP is now a concern for clinical counseling and decision making in reproductive-age women.

Limited reports to explore risk factors of polyp recurrence are available, mostly focused on postmenopausal women [1]. Only a few studies conducted in reproductive-age women have suggested some risk factors, such as the duration of follow-up, the number and size of polyps, and sex hormone therapy [2–4, 6]. Most of the abovementioned risk factors are related to proliferation potential of endometrial cells. Thus, the biomarkers for hyperplasia of endometrial squamous cells should be further studied.

The highly conserved RNA-binding protein LIN28 plays an important role in cell proliferation [7]. Human produce two LIN28 paralogs, LIN28A and LIN28B, which have been shown to be genetically associated with many hyperplastic diseases [8, 9]. Compared to LIN28A, LIN28B is a key regulator of the proliferation of endometrial squamous cells [10]. Therefore, *LIN28B* gene was analyzed in this study. Human *LIN28B* gene (HGNC: 32207) spans 14.6 kb on chromosome 6q16.3-q21, contains seven exons, and encodes a 250aa RNA-binding protein LIN28B. LIN28B negatively regulates pre-miRNA processing (GO: 2000632), including let-7 family, miR107, miR-143, and miR-200c [11]. It binds these pre-miRNAs and sequesters them away from the microprocessor complex, hence prevents them mature [11]. The overexpression of LIN28B is seen in various primary tumors, linked to the repression of let-7 family of microRNAs and derepression of let-7 targets, which facilitates cell proliferation [12–14]. Additionally, there were adequate researches about the effect of LIN28B on tumor recurrence as well. The aberrant expression of LIN28B is related to the recurrence of Wilms tumor [15], hepatocellular carcinoma [16], colon cancer [17], ovarian cancer [18], adrenocortical cancer, and so on [19, 20]. Moreover, *LIN28B* polymorphisms are significantly associated with recurrence of colorectal cancer [21]. However, no studies have reported the association between *LIN28B* polymorphisms and polyp recurrence after polypectomy. We hypothesized that genetic variants of *LIN28B* gene were associated with postoperative recurrence risk in EP patients.

In this hospital-based study conducted in a southern Chinese population between 2018 and 2021, we genotyped rs369065 C > T and rs314280 A > G polymorphisms in 351 reproductive female patients underwent hysteroscopic polypectomies and analyzed their associations with the risk of postoperative recurrence.

## 2. Methods

### 2.1. Study Population

This study is a genetic association study based on a hospital cohort, conducted in accordance with the reporting guidelines and checklist of Extension for Genetic Association Studies (STREGA) (Supplementary Table 1**)** [22]. In this study, 351 EP patients aged 20~ 40 years routinely diagnosed by hysteroscopy, ultrasonic imaging, and pathological examinations were enrolled at the Department of Reproductive Health in Shenzhen Baoan Women's and Children's Hospital of Jinan University between May 2018 and Jan 2020. We excluded patients with previous polypectomy, or any form of diffused endometrial hyperplasia. To improve or reserve the fertility, all these EP patients have undergone hysteroscopic polypectomy under sedation or intradural anesthetic, following the routine procedure of our hospital.

As for follow-up, all enrolled patients were scheduled to come back for routine examinations every six months after polypectomy, up until Jun 2021. Because all these patients have the intention to future pregnancy, no loss to follow-up has been reported in this study. The recurrence status was verified by ultrasonic imaging and pathological examinations according to two criteria: the same type of histology and the same location, which differentiates it from a new polyp [5]. We collected the patients' information from their electronic medical records, including age, menarche age, menses, parity, gravidity, dysmenorrhea, abnormal uterine bleeding, endometritis, pelvic infection, salpingitis, endometriosis, intrauterine adhesion, and blood analysis before polypectomy, the number, size and location of polyps, endometrial thickness, type of hysteroscopic polypectomy, and follow-up duration (disease-free interval until symptomatic recurrence). 5 ml of heparin anticoagulant blood of all enrolled patients was collected to perform genotyping. The participants signed a consent statement of ethical approval to use their samples and data.

This study was approved by the Ethics Committee of Shenzhen Baoan Women's and Children's Hospital, Jinan University (IRB No: LLSC-2018-08-01).

### 2.2. Single-Nucleotide Polymorphism Selection and Genotyping

We selected the single-nucleotide polymorphisms (SNPs) in *LIN28B* gene by using the dbSNP database according to the following three criteria [23]: (1) the minor allele frequencies (MAF) reported in HapMap database (http://hapmap.ncbi.nlm.nih.gov/) were more than 20% for Chinese Han subjects; (2) SNPs in low linkage disequilibrium with each other (*R*^2^ < 0.8). Based on these criteria, two SNPs rs369065 C > T and rs314280 A > G were selected in the present study.

Genomic DNA was extracted from blood samples using the MagaBio plus Blood DNA Kit (Bioer Technology, Hangzhou, China) according to the manufacturer's instructions. As described previously [23], genotyping of the abovementioned SNPs using Agenda Massarray technique was performed at CapitalBio Technology (Beijing, China), according to the manufacturer's protocol available at (doi:10.1007/978-1-4939-6442-0_5). Briefly, the process involved a locus-specific PCR reaction and a single-base extension reaction. The PCR products were purified by resin and added into a SpectroCHIP (Agena Bioscience, Sequenom, San Diego, California, USA), then were analyzed by MALDI-TOF techniques. As shown in Supplementary Figure 1, the call of genotyping was conducted by TYPER 4.0 (SEQUNOM). Genotyping was repeated on a random ~20% (70/351) of the samples, and the results were 100% concordant. All genotyping data of rs369065 C > T and rs314280 A > G polymorphisms were used for further analyses.

### 2.3. Statistical Analysis

To avoid bias of data collection, the data used for analyses were double-checked from the original medical records by two different research assistants. Missing data are very common in clinical researches, however, they were observed in the present study to a considerably low percentage (<5% of blood analyses and menstrual cycle/duration) and were replaced by the mean value of the variables. Chi-square test and Student's *t* test were used to compare the differences between recurrent and nonrecurrent patients regarding demographic characteristics. The Chi-Square goodness of fit test was applied to indicate whether the genotype frequency distribution of each polymorphism in all subjects under study was in Hardy–Weinberg equilibrium. The polyp recurrence rate and median recurrence time between different genotype groups were analyzed by Kaplan-Meier Survival Curve, log-rank test, and multiple Cox regression analysis by adjusting for age, gravidities, menarche age, the number and size of polyps, and endometrial thickness. The cut-off values for the abovementioned confounding factors were determined by using X-tile v3.6.1 (Yale University) [24]. Further stratification analyses by age, gravidities, circulating hemoglobin level, the number and size of polyps, and endometrial thickness were performed as well. The study size and statistical power were calculated using the PASS 15.0 software (NCSS LLC., Kaysville, Utah, USA). And the false-positive report probability (FPRP) test was applied to detect the false-positive association findings. All statistical analyses were performed using SPSS 18.0 software (IBM, Armonk, NY, USA). A two-sided statistical significance level of 0.05 was chosen as well.

## 3. Results

### 3.1. Characteristics of the Study Population

The average follow-up time was 801.5 ± 224.7 days. Until Jun 2021, there were 44 patients recurrent (12.5%). The distribution of demographic characteristics of patients is shown in Table 1. Overall, the differences in distributions of age, menses, parity, gravidity, dysmenorrhea, abnormal uterine bleeding, endometritis, pelvic infection, salpingitis, endometriosis, intrauterine adhesion, polyps' number, and endometrial thickness between the recurrent and nonrecurrent patients were not statistically significant (all *P* values > 0.05). However, the recurrent patients were more probably to have a lower level of circulating hemoglobin and have bigger polyps than were nonrecurrent patients (*P* values were 0.019 and 0.013, respectively) (Table 1). Therefore, these two variables, combined with other potential risk factors for polyp recurrence, were further adjusted for in the multivariate Cox regression model to control possible confounding on the main effects of the study polymorphisms. Additionally, they were used in later stratification analysis.

### 3.2. Association of LIN28B Polymorphisms with Postoperative Recurrence Risk in EP

The genotype distributions of the *LIN28B*rs369065 C > T and rs314280 A > G polymorphisms among the recurrent and non-recurrent patients are summarized in Table 2. The observed genotype frequencies of these two polymorphisms were all in agreement with the Hardy–Weinberg equilibrium in the study population (*P* values were 0.576 and 0.974, respectively). To control the statistical bias, we adjusted confounding factors in multiple Cox model using age, gravidities, menarche age, the size and number of polyps, and endometrial thickness.

As shown in Table 2, rs369065 C > T polymorphism was significantly associated with recurrence risk in a recessive genetic model: compared to the rs369065 CC/CT genotypes, carriers of TT genotype had an increased risk of polyp recurrence (adjusted hazard ratio [HR] = 1.883, 95% confidence interval [CI] = 1.033 − 3.434, *P* = 0.039). However, for the rs314280 A > G polymorphism, there was no significant association between this polymorphism and recurrence risk in either of genetic models.

**Figure 1:**
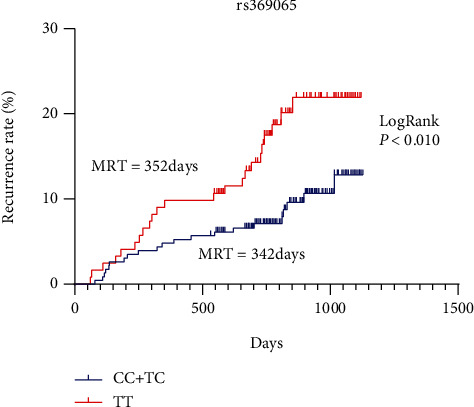
Kaplan-Meier survival curve for polyp recurrence by *LIN28B*rs369065 C > T polymorphism.

Further Kaplan-Meier survival curve and log-rank test showed that carries of rs369065 TT genotype had a shorter time to recurrence, compared with the CC/CT genotype (median time 352 vs. 342 days, log-rank *P* < 0.01) (Figure 1).

### 3.3. Stratification Analysis of LIN28B rs369065 TT Genotype and Risk of Polyp Recurrence

We further performed a stratification analysis of the associations between *LIN28B* rs369065 TT genotypes and risk of polyp recurrence by subgroups of age, gravidities, circulating hemoglobin level, the number and size of polyps, and endometrial thickness. As shown in Table 3, the increased risks of polyp recurrence associated with the rs369065 TT genotypes did not differ by gravidities, circulating hemoglobin level, and endometrial thickness (*P* > 0.05 for all subgroups). However, the harmful role of rs369065 TT genotypes in recurrence risk was more evident in the subjects with an older age (>33 years, adjusted HR = 2.597, 95%CI = 1.037 − 6.505, *P* = 0.042), a single polyp (adjusted HR = 2.545, 95%CI = 1.059 − 6.113, *P* = 0.037), and a smaller polyp (<1.2 cm, adjusted HR = 2.708, 95%CI = 1.042 − 7.043, *P* = 0.041). For the rs314280 A > G polymorphism, no significant association was found in any subgroup (data not shown).

## 4. Discussion

In this study, we found that rs369065 TT genotype of *LIN28B* gene was associated with an increased postoperative recurrence risk of EP in reproductive-age women, and the deleterious role of rs369065 TT genotype in recurrence risk was more evident in the subgroups older than 33 years, and those with fewer and smaller polyps.

Although LIN28B is a key factor for the etiology of occurrence [12, 25, 26] and recurrence [15, 18, 20] for many hyperplastic diseases, no literature has analyzed its role in the occurrence and recurrence of EP yet to our knowledge. In one of our previous studies conducted in 351 EP patients and 493 EP-free controls in a reproductive women population in South China, we did not find significant associations between *LIN28B* polymorphisms and the occurrence of EP (data not reported). However, we found that *LIN28B*rs369065 C > T polymorphism was significantly associated with an increased risk of polyp recurrence in this study. Through LIN28b/let-7 axis, *LIN28B* gene regulates the cell proliferation of endometrial stroma [10, 13, 27–29], which is tightly linked with the recurrence of EP. Taken together with our data and above reports, our findings on the association between *LIN28B* polymorphisms and polyp recurrence are plausible.

**Figure 2:**
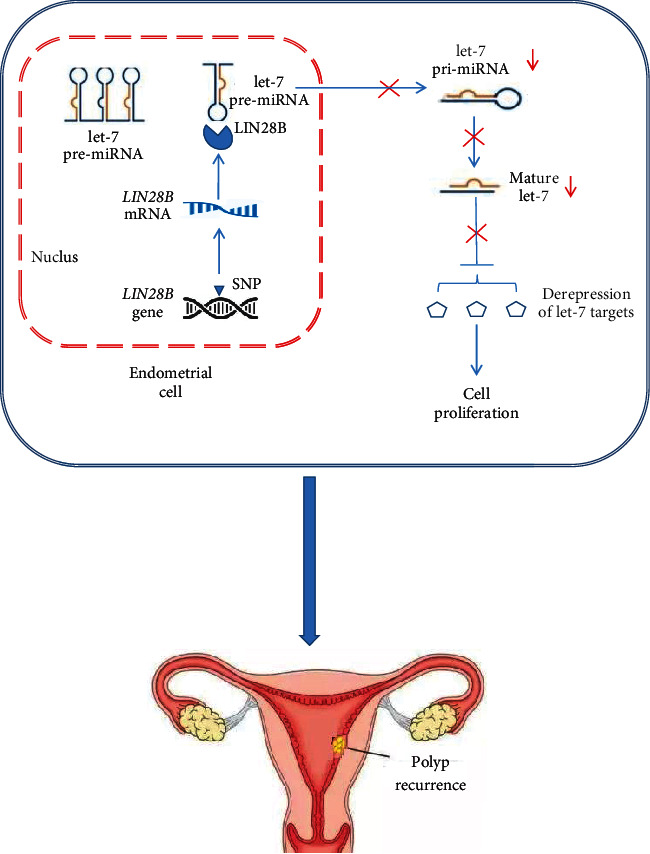
Potential mechanism of *LIN28B* polymorphisms in the recurrence of endometrial polyps.

A few population-based studies have analyzed the association of *LIN28B* polymorphisms with various phenotypes. They showed that *LIN28B*rs369065 C > T polymorphism is significantly associated with chronic hepatitis B virus infection [30], standing height [31], and age at menarche [32, 33]. These reports implied that *LIN28B*rs369065 C > T might be a potential functional polymorphism, which can alter *LIN28B* gene's expression or function, then promote the proliferation of endometrial squamous cells, thus, risk of polyp recurrence. However, there is another possible explanation: *LIN28B*rs369065 C > T polymorphism is tightly linked with another functional polymorphism rs221634 T > A in Chinese (*R*^2^ > 0.9). The substitution T > A of rs221634 causes losing a binding site of hsa-mir-548 family in the 3′ UTR of human *LIN28B* gene, as analyzed by SNPinfo (https://manticore.niehs.nih.gov/). The hsa-mir-548 family is highly expressed in human reproductive tissues [34] and is a pivotal suppressor of cell growth [35–37]. So we can also explain our results by that rs221634 T > A polymorphism is the real genetic cause of polyp recurrence. Combined with the abovementioned analyses, our findings on the association between *LIN28B* polymorphisms and polyp recurrence are biological creditable. As shown in Figure 2, the polymorphisms of *LIN28B* gene will affect the gene's expression and then affect the binding of LIN28B with let-7 pre-miRNA, preventing them mature, and causing derepression of let-7 targets, thus risk of polyp recurrence.

In the subgroup analysis, we found that the deleterious role of rs369065 TT genotype was absent in the patients with more and bigger polyps, who are known as susceptible population for polyp recurrence [3, 38]. The size and number of polyps relate to the cell proliferation of endometrial squamous cells, which is regulated by LIN28B/let-7 pathway [10]. This implies that the contribution of rs369065 C > T polymorphism to polyp recurrence could be modulated by environmental risk factors. In contrast, patients with fewer and smaller polyps, but carrying the harmful rs369065 TT genotype, still have an increased recurrence risk in this study. These findings suggest that a more precise choice should be made in clinical counseling and decision making for EP treatment.

This study had some limitations because it was a hospital-based cohort study, restricted to a Chinese Han population. However, the genotype frequencies of the two study polymorphisms among our subjects well fit the Hardy–Weinberg disequilibrium law, and T allele frequency of rs369065 in our study (61.1%) is similar to the frequencies reported in two studies conducted in two Chinese populations (64% [30] and 67% [39]), suggesting the subjects' selection is random. Additionally, we had achieved a more than 99% study power (two-sided test, *α* = 0.05) to detect a HR of 1.883 for the rs369065 TT genotype (which occurred at a frequency of 32.2% in the nonrecurrence subjects) compared with the rs369065 CC/CT genotypes in a population of more than 300 subjects. Therefore, it appears that our finding that the rs369065 TT genotype associated with an increased risk of polyp recurrence is unlikely to have been by chance. Also, when we performed the FPRP analysis and found that under the assumption of a prior probability of 0.05 and a prior odds ratio of 1.5 as suggested by Wacholder et al. [40], the FPRP for the observed association between the rs369065 C > T polymorphism and risk of polyp recurrence yielded a value of 0.165, which was lower than the pre-set FPRP-level criterion 0.20, suggesting that this finding is noteworthy.

## 5. Conclusions

For the first time, we found that *LIN28B*rs369065 C > T polymorphism was associated with an increased postoperative recurrence risk in reproductive-age women with EP, especially in some “low-risk” subpopulations, who had fewer and smaller polyps, implying a more precise choice of clinical counseling and decision making for EP patients. Larger studies in different ethnic populations are warranted.

## Figures and Tables

**Table 1:** Clinical and demographic characteristics of endometrial polyps patients with postoperative recurrence and nonrecurrence.

Variables	Recurrence, *n* (%)	Nonrecurrence, *n* (%)	*P* ^a^
All subjects	44 (100.0)	307 (100.0)	
Age, years			
<33	23 (52.3)	184 (59.9)	0.352
≥33	21 (47.7)	123 (40.1)	
Menarche age (years)			
<13	2 (4.5)	27 (8.8)	0.506
≥13	42 (95.5)	280 (91.2)	
Menstrual cycle (days)			
23-35	40 (90.9)	264 (86.0)	0.305
>35	4 (9.1)	43 (14.0)	
Menstrual duration (days)			
≤7	33 (75.0)	277 (90.2)	0.934
>7	11 (25.0)	30 (9.8)	
Dysmenorrhea	6 (13.6)	61 (19.9)	0.325
Abnormal uterine bleeding	4 (9.1)	10 (3.3)	0.151
Endometritis	16 (36.4)	101 (32.8)	0.648
Pelvic infection	2 (4.5)	7 (2.3)	0.705
Salpingitis	0 (0.0)	10 (3.3)	0.465
Endomotriosis	1 (2.3)	5 (1.6)	0.754
Intrauterine adhesion	1 (2.3)	7 (2.3)	0.591
Gravidities			
0	16 (36.4)	126 (41.0)	0.470
1	12 (27.3)	81 (26.4)	
≥2	16 (36.4)	100 (32.6)	
Abortion			
0	28 (63.6)	208 (67.8)	0.610
1	11 (25.0)	66 (21.5)	
≥2	5 (11.4)	33 (10.8)	
Deliveries			
0	20 (45.5)	164 (53.4)	0.429
1	15 (34.1)	82 (26.7)	
≥2	9 (20.5)	61 (19.9)	
Red blood cells (×10^12^/L)			
<4.37	23 (52.3)	156 (50.8)	0.602
≥4.37	21 (47.7)	151 (49.2)	
Hemoglobin (g/L)			
<126	28 (63.64)	146 (47.56)	0.019
≥126	16 (36.36)	161 (52.44)	
White blood cells (×10^9^/L)			
<6.09	25 (56.82)	150 (48.86)	0.206
≥6.09	19 (43.18)	157 (51.14)	
Number of polyps			
Single polyp	21 (47.73)	196 (63.84)	0.059
Multiple polyps	23 (52.27)	111 (36.16)	
Diameter of polyps (cm)			
<1.2	18 (40.9)	210 (68.4)	0.013
≥1.2	26 (59.1)	97 (31.6)	
Endometrial thickness (cm)			
<0.9	22 (50.0)	153 (49.2)	0.984
≥0.9	22 (50.0)	154 (50.8)	
Average follow-up days	801.5 ± 224.7		

Notes: ^a^Chi-square tests or Fisher exact test.

**Table 2:** The associations between *LIN28B* gene polymorphisms and recurrence of EP.

Genotypes	Recurrence, *n* (%)	Nonrecurrence, *n* (%)	*P* for HWP^a^	Adjusted HR (95% CI)^b^	*P* for Cox model
Subjects	44	307			
rs369065 (C > T)					
CC	5 (11.4)	48 (15.6)	0.576	Ref. (1.00)	
CT	16 (36.4)	160 (51.1)		1.115 (0.406-3.061)	0.833
TT	23 (52.3)	99 (32.2)		2.044 (0.768-5.442)	0.152
Dominant	39 (88.6)	259 (84.4)		1.512 (0.591-3.867)	0.388
Recessive	21 (47.7)	208 (67.8)		1.883 (1.033-3.434)	0.039
rs314280 (A > G)					
AA	3 (6.8)	30 (9.8)	0.974	Ref. (1.00)	
GA	13 (29.5)	138 (45.0)		0.904 (0.249-3.275)	0.878
GG	28 (63.6)	139 (45.3)		1.613 (0.472-5.5.7)	0.446
Dominant	41 (93.2)	277 (90.2)		1.271 (0.382-4.234)	0.696
Recessive	16 (36.4)	168 (54.7)		1.751 (0.916-3.349)	0.090

^a^The observed genotype frequency in the study population was in agreement with the Hardy–Weinberg equilibrium (*p*^2^ + 2pq + *p*^2^ = 1) (*χ*^2^ = 1.103, *P* = 0. 576 for rs369065; *χ*^2^ = 0.053, *P* = 0.974 for rs314280). HR: hazard ratio. ^b^Adjusted age, gravidities, menarche age, number and size of polyps, and endometrial thickness.

**Table 3:** Stratification analysis of *LIN28B* rs369065 polymorphism by selected variables in recurrent and nonrecurrent patients.

	Recurrence (*n* = 44)		Nonrecurrence (*n* = 307)		Adjusted HR (95% CI)^a^	*P*
	CC/TC	TT	CC/CT	TT		
	*n* (%)	*n* (%)	*n* (%)	*n* (%)		
Age (years)						
<33	13 (56.5)	10 (43.5)	121 (65.8)	63 (34.2)	1.300 (0.559-3.022)	0.542
≥33	8 (38.1)	13 (61.9)	87 (70.7)	36 (29.3)	2.597 (1.037-6.505)	0.042
Gravidities						
0	7 (43.8)	9 (56.2)	78 (60.9)	48 (39.1)	1.565 (0.570-4.299)	0.385
1	6 (50.0)	6 (50.0)	61 (75.3)	20 (24.7)	1.886 (0.556-6.402)	0.309
≥2	8 (50.0)	8 (50.0)	69 (69.0)	31 (31.0)	1.914 (0.708-5.173)	0.201
Hemoglobin (g/L)						
<126	14 (31.82)	14 (31.82)	95 (30.94)	51 (16.61)	1.837 (0.850-3.973)	0.122
≥126	7 (15.91)	9 (20.45)	113 (36.81)	48 (15.64)	1.862 (0.657-5.275)	0.242
Number of polyps						
Single polyp	10 (47.6)	11 (52.4)	139 (70.9)	57 (29.1)	2.545 (1.059-6.113)	0.037
Multiple polyps	11 (47.8)	12 (52.2)	69 (62.2)	42 (37.8)	1.570 (0.683-3.611)	0.288
Diameter of polyp (cm)						
<1.2	18 (47.4)	20 (52.6)	170 (69.4)	85 (30.6)	2.708 (1.042-7.043)	0.041
≥1.2	3 (50.0)	3 (50.0)	38 (73.1)	14 (26.9)	1.458 (0.672-3.165)	0.340
Endometrial thickness (cm)						
<0.9	10 (45.5)	12 (54.5)	102 (66.7)	51 (33.3)	2.189 (0.920-5.209)	0.077
≥0.9	11 (50.0)	11 (50.0)	106 (68.8)	48 (31.2)	1.774 (0.750-4.200)	0.192

HR: hazard ratio. ^a^Adjusted age, gravidities, menarche age, number and size of polyps, and endometrial thickness.

## Data Availability

The raw data supporting the conclusions of this article will be made available by the authors, without undue reservation.

## Supplementary Materials

Supplementary 1 Supplementary Figure 1: Cartesian plots of rs369065 C > T and rs314280 A > G polymorphisms analyzed using Agenda MassArray technique.

Supplementary 2 Supplementary Table 1: STREGA checklist for this study.
