# Supplementary figures and images for: LIN28B Polymorphisms Confer a Higher Postoperative Recurrence Risk in Reproductive-Age Women with Endometrial Polyps
Source: Dis Markers. 2022 Feb 27;2022:4824357. doi: 10.1155/2022/4824357 (PMC8902632; doi:10.1155/2022/4824357)

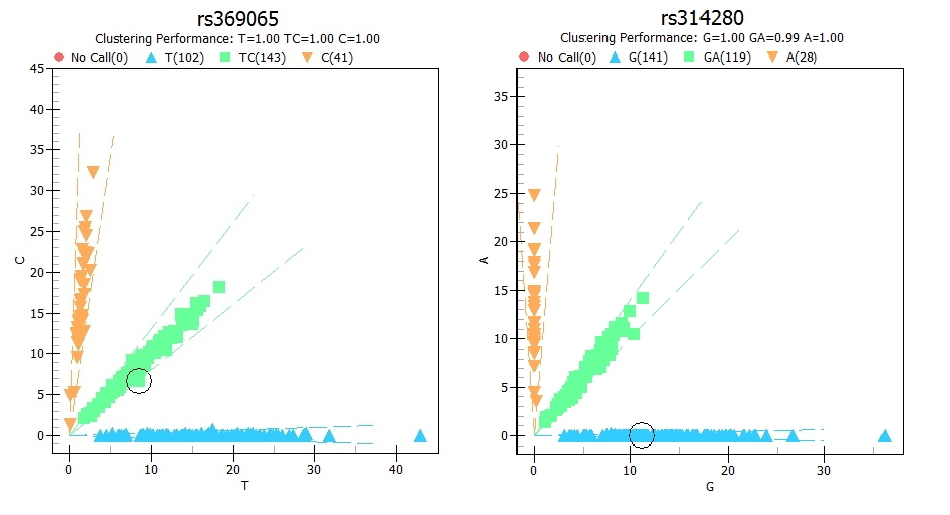

Supplement: Supplementary 1 — Supplementary Figure 1: Cartesian plots of rs369065 C > T and rs314280 A > G polymorphisms analyzed using Agenda MassArray technique. [file 4824357.f1.png]
