# Supplementary material for: LIN28B Polymorphisms Confer a Higher Postoperative Recurrence Risk in Reproductive-Age Women with Endometrial Polyps
Source: Dis Markers. 2022 Feb 27;2022:4824357. doi: 10.1155/2022/4824357 (PMC8902632; doi:10.1155/2022/4824357)
Supplement: Supplementary 2 — Supplementary Table 1: STREGA checklist for this study. [file 4824357.f2.doc]

# Supplementary TABLE 1. STREGA checklist

| **Item** | **Item number** | **STROBE Guideline** | **Extension for Genetic Association Studies (STREGA)** | **Page** |
| --- | --- | --- | --- | --- |
| **Title and Abstract** | 1 | (a) Indicate the study’s design with a commonly used term in the title or the abstract. |  | In the abstract, Page 2 |
|  |  |  |  |  |
| **Introduction** | | |  |  |
| *Background rationale* | 2 | Explain the scientific background and rationale for the investigation being reported. |  | Page 3 |
| *Objectives* | 3 | State specific objectives, including any pre-specified hypotheses. | ***State if the study is the first report of a genetic association, a replication effort, or both.*** | Page 4  ***is the first report*** |
| **Methods** | | |  |  |
| *Study design* | 4 | Present key elements of study design early in the paper. |  | Page 4 |
| *Setting* | 5 | Describe the setting, locations and relevant dates, including periods of recruitment, exposure, follow-up, and data collection. |  | Page 4 |
| *Participants* | 6 | 1. **Cohort study –** Give the eligibility criteria, and the sources and methods of selection of participants. Describe methods of follow-up.   **Case-control study –** Give the eligibility criteria, and the sources and methods of case ascertainment and control selection. Give the rationale for the choice of cases and controls.  **Cross-sectional study –** Give the eligibility criteria, and the sources and methods of selection of participants. | ***Give information on the criteria and methods for selection of subsets of participants from a larger study, when relevant***. | Eligibility criteria, selection of patients, methods of follow-up were described in **Page 4**. Not from a larger study. |
|  |  | 1. **Cohort study –** For matched studies, give matching criteria and number of exposed and unexposed.   **Case-control study –** For matched studies, give matching criteria and the number of controls per case. |  | Not applicable,Not a matched study. |
| *Variables* | 7 | *(a)* Clearly define all outcomes, exposures, predictors, potential confounders, and effect modifiers. Give diagnostic criteria, if applicable. | ***(b)*** ***Clearly define genetic exposures (genetic variants) using a widely-used nomenclature system. Identify variables likely to be associated with population stratification (confounding by ethnic origin).*** | Yes, from dbSNP database, identified SNPs from Chinese.  Page 5 |
| *Data sources measurement* | 8***** | *(a)* For each variable of interest, give sources of data and details of methods of assessment (measurement). Describe comparability of assessment methods if there is more than one group. | ***(b)*** ***Describe laboratory methods, including source and storage of DNA, genotyping methods and platforms (including the allele calling algorithm used, and its version), error rates and call rates. State the laboratory/centre where genotyping was done****.* ***Describe comparability of laboratory methods if there is more than one group. Specify whether genotypes were assigned using all of the data from the study simultaneously or in smaller batches.*** | Page 5,  Including genotyping methods and platforms,call rates,centre where genotyping was done. All data were assigned using. |
| *Bias* | 9 | *(a)* Describe any efforts to address potential sources of bias. | ***(b) For quantitative outcome variables, specify if any investigation of potential bias resulting from pharmacotherapy was undertaken. If relevant, describe the nature and magnitude of the potential bias, and explain what approach was used to deal with this.*** | No quantitative outcome variables, not for pharmacotherapy |
| *Study size* | 10 | Explain how the study size was arrived at. |  | Using PS software, Page 6 |
| *Quantitative variables* | 11 | Explain how quantitative variables were handled in the analyses. If applicable, describe which groupings were chosen, and why. | ***If applicable, describe how effects of treatment were dealt with.*** | The cut-off values were set by X-title software, Page 6 |
| Statistical methods | 12 | (a) Describe all statistical methods, including those used to control for confounding. | ***State software version used and options (or settings) chosen.*** | software version in Page 6 |
|  |  | (b) Describe any methods used to examine subgroups and interactions. |  | stratification analyses, Page 6 |
|  |  | (c) Explain how missing data were addressed. |  | Replace by the mean, Page 5 |
|  |  | 1. **Cohort study –** If applicable, explain how loss to follow-up was addressed.   **Case-control study –** If applicable, explain how matching of cases and controls was addressed.  **Cross-sectional study –** If applicable, describe analytical methods taking account of sampling strategy. |  | No loss for this study, because all pateients have the intention to future pregnancy, willing back to hospital  Page 4 |
|  |  | (e) Describe any sensitivity analyses. |  | Not applicable |
|  |  |  | ***(f) State whether Hardy-Weinberg equilibrium was considered and, if so, how****.* | Yes, Line 128 (Page 5) |
|  |  |  | ***(g) Describe any methods used for inferring genotypes or haplotypes.*** |  |
|  |  |  | ***(h) Describe any methods used to assess or address population stratification.*** |  |
|  |  |  | ***(i) Describe any methods used to address multiple comparisons or to control risk of false positive findings.*** |  |
|  |  |  | ***(j) Describe any methods used to address and correct for relatedness among subjects*** |  |
| **Results** | | |  |  |
| *Participants* | 13***** | 1. Report the numbers of individuals at each stage of the study – e.g., numbers potentially eligible, examined for eligibility, confirmed eligible, included in the study, completing follow-up, and analysed. | ***Report numbers of individuals in whom genotyping was attempted and numbers of individuals in whom genotyping was successful.*** | Table 2  (all seccessful) |
|  |  | (b) Give reasons for non-participation at each stage. |  | Not applicable |
|  |  | (c) Consider use of a flow diagram. |  | Not applicable |
| *Descriptive data* | 14***** | (a) Give characteristics of study participants (e.g., demographic, clinical, social) and information on exposures and potential confounders. | ***Consider giving information by genotype****.* |  |
|  |  | (b) Indicate the number of participants with missing data for each variable of interest. |  | Not applicable |
|  |  | 1. **Cohort study –** Summarize follow-up time, e.g. average and total amount. |  | Yes, Table 1 |
| *Outcome data* | 15 ***** | **Cohort study-**Report numbers of outcome events or summary measures over time. | ***Report outcomes (phenotypes) for each genotype category over time*** | Yes, Figure 1 |
|  |  | **Case-control study –** Report numbers in each exposure category, or summary measures of exposure. | ***Report numbers in each genotype category*** |  |
|  |  | **Cross-sectional study –** Report numbers of outcome events or summary measures. | ***Report outcomes (phenotypes) for each genotype category*** | ***Not applicable*** |
| *Main results* | 16 | (a) Give unadjusted estimates and, if applicable, confounder-adjusted estimates and their precision (e.g., 95% confidence intervals). Make clear which confounders were adjusted for and why they were included. |  | Table 2 |
|  |  | (b) Report category boundaries when continuous variables were categorized. |  | Table 2 |
|  |  | (c) If relevant, consider translating estimates of relative risk into absolute risk for a meaningful time period. |  | Not applicable |
|  |  |  | ***(d) Report results of any adjustments for multiple comparisons.*** | Just 2 SNPs， no need for Bonferroni;  But statistical power were discussed in ***Discussion*** |
| *Other analyses* | 17 | 1. Report other analyses done – e.g., analyses of subgroups and interactions, and sensitivity analyses. |  | Yes,  Table 3 |
|  |  |  | ***(b) If numerous genetic exposures (genetic variants) were examined, summarize results from all analyses undertaken.*** | ***Not applicable*** |
|  |  |  | ***(c) If detailed results are available elsewhere, state how they can be accessed.*** | ***Not applicable*** |
| **Discussion** | | |  |  |
| *Key results* | 18 | Summarize key results with reference to study objectives. |  | Page 7 |
| *Limitations* | 19 | Discuss limitations of the study, taking into account sources of potential bias or imprecision. Discuss both direction and magnitude of any potential bias. |  | Page 9 |
| *Interpretation* | 20 | Give a cautious overall interpretation of results considering objectives, limitations, multiplicity of analyses, results from similar studies, and other relevant evidence. |  | Page 7-9 |
| *Generalizability* | 21 | Discuss the generalizability (external validity) of the study results. |  | In ***Conclusion***, Page 9 |
| **Other Information** | | |  |  |
| *Funding* | 22 | Give the source of funding and the role of the funders for the present study and, if applicable, for the original study on which the present article is based. |  | Page 10 |

STREGA = STrengthening the REporting of Genetic Association studies; STROBE = STtrengthening the Reporting of Observational Studies in Epidemiology.

* Give information separately for cases and controls in case-control studies and, if applicable, for exposed and unexposed groups in cohort and cross-sectional studies.
